# Maladaptive Perfectionism and Depression: Testing the Mediating Role of Self-Esteem and Internalized Shame in an Australian Domestic and Asian International University Sample

**DOI:** 10.3389/fpsyg.2020.01272

**Published:** 2020-06-10

**Authors:** Benjamin Dorevitch, Kimberly Buck, Matthew Fuller-Tyszkiewicz, Lisa Phillips, Isabel Krug

**Affiliations:** ^1^Melbourne School of Psychological Sciences, The University of Melbourne, Melbourne, VIC, Australia; ^2^Centre for Social and Early Emotional Development, School of Psychology, Deakin University, Melbourne, VIC, Australia; ^3^School of Psychology, Deakin University, Geelong, VIC, Australia

**Keywords:** depression, perfectionism, internalized shame, self-esteem, international students

## Abstract

**Objectives:**

To assess whether maladaptive perfectionism (parental and intrapersonal), mediated by self-esteem and internalized shame, lead to depressive symptoms, and to identify whether this model was invariant (i.e., structurally unchanged) across groups of Australian domestic and Asian international students.

**Methods:**

A total of 624 (308 Australian domestic and 316 Asian international undergraduate university students) completed a questionnaire on the variables of interest.

**Results:**

Australian domestic and Asian international students did not significantly differ in reported levels of study variables apart from parental maladaptive perfectionism, on which Australian domestic students scored significantly higher. The proposed path-model differed across student groups, with findings indicating that intrapersonal maladaptive perfectionism impacted indirectly on depressive symptoms through internalized shame in both groups, however, indirectly through self-esteem in only the Asian international student group.

**Conclusion:**

Intrapersonal maladaptive perfectionism may be a culturally independent process, capable of predisposing all university students to develop depressive symptoms, but that self-esteem may be a particularly relevant mediator of this relationship among Asian international students.

## Introduction

In 2016, over 24% of the 1.2 million higher education students studying in Australia were international students. An overwhelming proportion of these students originated from Asian countries (Department of Education and Training, 2017). Although commencing study at university can be stressful for almost all students, a range of acculturative stressors (e.g., language barriers, loss of family and social support, racial discrimination) can make this transition particularly challenging for Asian international students, who may be at high risk of developing depressive symptoms compared to their Australian domestic student counterparts (e.g., Schofield et al., 2016). In addition to acculturative stressors, specific dispositional characteristics may predispose Asian international students to depression more so than to local student (e.g., Wei et al., 2007; Yoon and Lau, 2008; Rice et al., 2012). One such predisposing factor is maladaptive perfectionism, which has been revealed to be especially pertinent to Asian international students (e.g., Yoon and Lau, 2008; Smith and Khawaja, 2011; Schofield et al., 2016). It is thought to exert effects on depression for both international and local students via other dispositional characteristics, including self-esteem (e.g., Shafran et al., 2002; Sowislo and Orth, 2013) and internalized shame (e.g., Ashby et al., 2006; Porter et al., 2018). However, these proposed pathways to depression have rarely been compared cross-culturally between groups of domestic Australian and Asian international students. This study aimed to test, for the first time in a mixed Australian domestic and Asian international university student sample, the utility of a proposed path-model of maladaptive perfectionism and depressive symptoms mediated by self-esteem and internalized shame.

### Depressive Symptoms in International Asian Students

Depressive symptoms have been consistently highlighted as a critical issue for Asian international students (e.g., Pan et al., 2007; Sawir et al., 2008; Smith and Khawaja, 2011; Schofield et al., 2016). For example, Sawir et al. (2008), conducted a large qualitative study interviewing 200 international students at different Australian universities, and found over two-thirds of the sample reported significant experiences of isolation and loneliness, with many respondents endorsing stronger negative mood such as depression. Similarly, in a sizeable correlational study conducted by Pan et al. (2007), 400 Chinese students from different universities in Hong Kong, and 227 Chinese students studying overseas at the University of Melbourne, were asked to complete a questionnaire including measures of acculturative stress and negative affect. Results showed that the Australian sample yielded significantly higher scores than their Hong Kong counterparts on the measure of negative affect (which included items such as feelings of sadness, helplessness, and depression), with the authors concluding that acculturative stressors facing international students may increase their vulnerability to developing depressive symptoms (Musiat et al., 2014). Conversely, a large correlational study conducted by Stallman (2010), in which online questionnaire data was collected from 6,479 students across two large Australian universities, revealed no significant differences in the levels of distress, or in the prevalence of anxiety and mood disorders; between international and domestic Australian tertiary students. However, with under 15% of the sample representing international students, and a low average response rate of 8.4% across the two universities; the representativeness of the sample in this study is questionable.

It is important to note here that many studies in this area have examined depression amongst international students in the context of variables such as acculturative stress (e.g., Smith and Khawaja, 2011) or level of social support (e.g., Sümer et al., 2008), which aim to explain the direct mechanism by which adverse mental health outcomes eventuate following a stressor. Other research (e.g., Rice et al., 2012) to which the current study aims to contribute – has aimed to investigate the personal or dispositional characteristics of international students that may serve to predispose, or create underlying *vulnerabilities* to developing later depression; such as perfectionism.

### Perfectionism as a Multidimensional Construct

Over the past few decades, many conceptualizations of perfectionism have been offered, with a substantial body of research supporting the view that perfectionism is a multidimensional construct; with both ‘normal’ and ‘neurotic/maladaptive’ subtypes (Hamachek, 1978; Frost et al., 1990; Flett and Hewitt, 2002; Stoeber and Otto, 2006; Rice and Ashby, 2007). Hamachek (1978) described normal perfectionism as consisting of a healthy pursuit of goals or high standards where, if their attainment were frustrated, a person would not suffer a loss of self-worth. Neurotic/maladaptive perfectionism, in contrast, is typified by the self-worth of a person being closely tied to their achievement of personal goals and a lack of satisfaction when a person succeeds in attaining them (Rice and Ashby, 2007).

Perfectionism has also often been referred to as self-oriented versus other-oriented versus socially prescribed perfectionism (Hewitt and Flett, 1991). Whereas self-oriented perfectionists obey to high achieving attributes for oneself, other-oriented perfectionists expect perfection from significant others (e.g., children, partners, co-workers) in different life and work domains. Finally, socially prescribed perfectionism is the tendency for an individual to believe that others expect perfection from that individual. This type of perfectionism, therefore, emerges from a fear of failure and/or a desire to avoid embarrassment, shame and guilt (Hewitt and Flett, 1991). Socially prescribed perfectionism has been most commonly linked to Hamachek’s (1978) constellation of neurotic perfectionism. Research has shown that self-oriented and socially prescribed perfectionism are the types of perfectionism, most commonly found to be associated with adverse health outcomes, such as depression (e.g., Hewitt and Flett, 1991; Hewitt et al., 1996) and eating disorders (e.g., Bardone-Cone, 2007).

One of the most widely used measures to assess the multifaceted features of perfectionism is the Multidimensional Perfectionism Scale (FMPS; Frost et al., 1990). Frost et al. (1990) initially identified six dimensions of perfectionism for the FMPS, four of which have since been associated with maladaptive outcomes (e.g., depression, anxiety, stress); namely Concern over Mistakes, Doubt about Actions, Parental Expectations and Parental Criticism (e.g., Stoeber, 1998). Factor analytic research on the FMPS (e.g., Stoeber, 1998; Purdon et al., 1999) has suggested the existence of two underlying factors; namely ‘intrapersonal’ maladaptive perfectionism (including Concern over Mistakes, and Doubts about Actions dimensions), and ‘parental’ maladaptive perfectionism (including Parental Expectations and Parental Criticism dimensions; Stoeber, 1998; Soenens et al., 2005; Stoeber and Otto, 2006). Relatively few studies have examined maladaptive perfectionism in terms of parental and intrapersonal subtypes. Soenens et al. (2005) investigated a Belgian sample of 171 psychology students and found that a composite score of intrapersonal perfectionism derived from the FMPS predicted higher levels of depression and lower self-esteem. In a later study by Yoon and Lau (2008) of 140 Asian American university students, intrapersonal and parental maladaptive perfectionism composite scores both strongly predicted depressive symptoms. Yoon and Lau (2008) suggested that these findings served as preliminary support for the assertion that distress stemming from concern with duty to fulfill ones’ parents’ expectations or avoid parental criticism, may be particularly relevant for students coming from an Asian cultural context (e.g., Chao and Tseng, 2002). However, as mentioned previously, maladaptive perfectionism has rarely been examined in terms of these two subtypes, and it has instead often been treated as a unitary construct (e.g., Lo and Abbott, 2013). Furthermore, despite a wealth of research establishing associations between maladaptive perfectionism and depression, to the authors’ knowledge none other than Yoon and Lau (2008), have attempted to compare the respective pathways of parental and maladaptive perfectionism with depressive symptoms, and none have done so while investigating domestic and Asian international student groups.

### Maladaptive Perfectionism and Depression

Across the literature, one finding with consistent support is that maladaptive perfectionism is strongly associated with higher levels of depressive symptoms (e.g., Egan et al., 2011). Many cross-sectional studies have shown various aspects of maladaptive perfectionism to be elevated in clinically depressed individuals compared to healthy controls (e.g., Sassaroli et al., 2008; Jacobs et al., 2009; Egan et al., 2011; Cheng et al., 2015), while longitudinal research has found maladaptive perfectionism to predict increases in depressive symptomatology over time in non-clinical samples (e.g., Smith et al., 2017). For example, in a 4-year prospective study investigating a community-based sample of 150 adults, Mandel et al. (2015) found self-critical perfectionism predicted the development of depressive symptoms after mediation by responses to stressors of daily living.

Research suggests that the relationship between maladaptive perfectionism and depressive symptomatology may be particularly pertinent for Asian international students (e.g., Wei et al., 2007; Nilsson et al., 2008; Yoon and Lau, 2008; Rice et al., 2012; Huang and Mussap, 2016). For example, Wei et al. (2007) conducted a cross-sectional study investigating 189 international students from China and Taiwan studying abroad in the U.S. at the time of the study and found that decreased maladaptive perfectionism moderated the effect of acculturative stress on depressive symptoms. The authors asserted that Asian international students displaying high maladaptive perfectionism might have interpreted the difficulties associated with studying abroad as a personal failure, increasing their vulnerability for developing depression (Wei et al., 2007). Similarly, in a recent cross-sectional Australian study utilizing a large sample of 384 Asian international students, Huang and Mussap (2016) found that lower levels of maladaptive perfectionism mitigated the effects of acculturative stress on depressive symptoms, with this effect more pronounced in international students who had spent more time in Australia.

These findings are not unanimous; some studies in this area (e.g., Hamamura and Laird, 2014) have yielded no differences between Asian international and domestic (North American) student groups on measures of maladaptive perfectionism and depressive symptoms. In addition, Khawaja and Dempsey (2007) found that maladaptive perfectionism did not predict variance on a measure of psychological distress (including items assessing depressive symptoms) in a sample of international students at an Australian university. However, both of these studies possessed fundamental limitations, investigating small international student samples (*n* = 52 and 86 respectively) and employing simple statistical methods (Pearson product-moment correlations and standard multiple regression) to test their hypotheses; limiting the validity of these findings. It is also important to note here, that most research in this area has endorsed the perspective of an indirect relationship between maladaptive perfectionism and depressive symptoms, asserting that it may be mediated by other mechanisms (e.g., Ashby et al., 2006; Noble et al., 2014). Two such potential mediating factors are self-esteem (e.g., Shafran et al., 2002) and internalized shame (e.g., Ashby et al., 2006).

### Mediators of Maladaptive Perfectionism and Depression

#### Self-Esteem

Low self-esteem has long been associated with depression, with longitudinal studies suggesting that negative self-evaluations constitute a causative risk and perpetuating factor for depressive symptoms (e.g., Sowislo and Orth, 2013). Research in the domain of perfectionism has subsequently examined and found support for the mediating role of self-esteem between maladaptive perfectionism and depression (e.g., Shafran et al., 2002). For example, early work with American college student samples found that low self-esteem mediated the relationship between maladaptive perfectionism and depressive symptoms (e.g., Flett et al., 2003). More recent cross-sectional studies replicated these findings in community-based adult samples (Moroz and Dunkley, 2015). These convergent findings suggest that maladaptive perfectionists may hold a contingent sense of self-worth, making them more vulnerable to experiencing psychological distress (e.g., depression), when they encounter negative events or perceptions of failure. However, to date, no research examining self-esteem as a possible mediating variable between maladaptive perfectionism and depression has classified maladaptive perfectionism in terms of parental and intrapersonal subtypes and compared the potential pathways between these variables across domestic and Asian international university student groups.

#### Internalized Shame

Shame has also been repeatedly linked to depressive symptoms (e.g., Kim et al., 2011), and has more specifically been implicated in the mental health problems experienced by Asian international tertiary students (e.g., Anolli and Pascucci, 2005; Smith and Khawaja, 2011). For instance, in an Italian study, Asian international students were found to experience stronger and more enduring shame than their Italian domestic student counterparts (Anolli and Pascucci, 2005). Sociological and anthropological studies suggest that internalized shame may be intrinsically linked with numerous Asian cultures, evidenced by a variety of words for types of shame in Asian languages, culturally accepted shame-based parenting techniques, and a collectivist sense of self that is inherently dependent on gaining the approval of others (e.g., Lutwak et al., 1998; Fung, 1999; Bedford, 2004).

Shame has also been linked to perfectionism (e.g., Wyatt and Gilbert, 1998); however, only one study to date has examined the potential mediational role of shame in the relationship between maladaptive perfectionism and depression. This study (Ashby et al., 2006), which was conducted in a sample of US college students, found that the relationship between maladaptive perfectionism and depression was mediated by both internalized shame and self-esteem. However, this finding was only observed amongst females, but not males. Again, no studies have yet explored these relationships among Asian international students, and none have examined potential cross-cultural differences in these relationships by comparing across groups of domestic and Asian international university students. Furthermore, no studies exploring these mediators have delineated the parental and intrapersonal subtypes of maladaptive perfectionism.

### Gaps in the Literature

Given that Asian international students constitute a ‘high risk’ group for developing depression (e.g., Schofield et al., 2016), there is a need for further research clarifying the influence of intrapersonal and parental perfectionism on depression within this student population to inform future interventions and support services. It would also be valuable to compare these relationships across domestic and Asian international student groups to shed light on the cross-cultural nature of the processes by which university students develop depression from different types of perfectionistic tendencies. Furthermore, although some studies have examined depressive symptoms amongst Asian international students in Australia (e.g., Pan et al., 2007; Sawir et al., 2008; Smith and Khawaja, 2011), few have done so incorporating data from students attending university psychology clinics (e.g., Raunic and Xenos, 2008). This is significant, as Asian international students may face numerous additional barriers to help-seeking compared to their domestic student counterparts; such as cultural perceptions of psychological problems as shameful or indicative of weakness, somatization of psychological distress, or systemic barriers (e.g., inability to access the public health system in Australia; Smith and Khawaja, 2011). Due to their proximity and affordability, university psychology clinics, therefore, form an essential support service for international students (Raunic and Xenos, 2008), and a vital source of data for furthering our understanding of psychological problems facing this population.

### The Current Study

The current study, therefore, aimed to address some of the abovementioned gaps in the literature by firstly examining reported levels of maladaptive perfectionism (both intrapersonal and parental subtypes), self-esteem, internalized shame, and depressive symptoms across Australian domestic and Asian international student groups to identify potential cross-cultural differences. The second aim was to examine relationships between study variables in the student groups by assessing the utility of a proposed path model, in which maladaptive perfectionism, mediated by self-esteem and internalized shame, led to depressive symptoms, and to assess whether this model was invariant across Australian domestic and Asian international students.

To ensure an adequate range of depression severity, the current sample comprised of both non-clinical university students and attendees of the University’s Psychology Clinic. Data from the whole sample (i.e., both ‘clinical’ and ‘non-clinical’ students) were included in all analyses. However, this raises the possibility that recruitment strategy (via university vs. via psychology clinic) may be associated with other variables in our model. As such, we include recruitment type (1 = clinic, 0 = university) as a covariate in our model. Participants’ age and gender were also controlled for all analyses, as both factors are known to contribute significant variability to the presentation of depressive symptomatology amongst university students (Ibrahim et al., 2013).

It was hypothesized that: (1) Asian international students would have higher levels of depressive symptoms, parental maladaptive perfectionism, and internalized shame compared to domestic Australian students. Due to inconclusive data on cross-cultural differences in self-esteem (e.g., Schmitt and Allik, 2005), no differences were predicted between the student groups; (2) higher intrapersonal and parental maladaptive perfectionism, lower self-esteem, and higher internalized shame would be significantly associated with higher depressive symptoms for both student groups; and (3) the proposed path model would differ (i.e., not invariant) across both student groups.

## Materials and Methods

### Participants

Participants were university students recruited either through an undergraduate psychology research experience program or via the University Psychology Clinic, where the study took part. The final sample (*n* = 624) comprised of 35 clinic attendees, of which 23 were domestic and 12 were Asian international students; and 589 non-clinical undergraduate students, of which 285 were Australian domestic and 304 were Asian international students. Asian international students were defined by international fee-paying student status, and ethnicity indicated. Thirteen students from the research experience program reported that they had previously utilized psychological services at either the University Psychology Clinic or the Counselling Service from the same university; however, these individuals were deemed to be ‘non-clinical’ cases for this study as they were not currently seeking those services.

### Measures

#### Demographics

Participants provided information about their age, sex, ethnic/racial background; years lived in Australia, university fee-paying status (domestic or international), and history of psychological service utilization.

#### Depression, Anxiety and Stress Scales (DASS-21)

The DASS-21 (Lovibond and Lovibond, 1995) is designed to assess the presence of depression, anxiety, and stress symptoms. Participants indicated the extent to which 21 statements (e.g., *“I felt down-hearted and blue”*) applied to them in the past week on a 4-point response scale from 1 (*never*) to 4 (*almost always*). Only items relating to depressive symptoms (seven items) were analyzed. Scores were summed to give a total score ranging from 7 to 28, with higher scores indicating greater depressive symptom severity (Lovibond and Lovibond, 1995). The DASS-21 had good reliability in the current sample (Cronbach’s α = 0.92) and has previously demonstrated good internal consistency and construct validity (Lovibond and Lovibond, 1995).

#### Frost Multidimensional Perfectionism Questionnaire (FMPS)

The FMPS (Frost et al., 1990) assesses adaptive and maladaptive forms of perfectionism. Participants responded to 35 personal statements (e.g., “I should be upset if I make a mistake”) belonging to six subscales: *concern over mistakes* (CM; nine items), *parental expectations* (PE; five items), *parental criticism* (PC; four items), *doubt about action*, (DA; four items), *personal standards* (PS; seven items) and *organization* (O; six items). Items were rated on a 5-point Likert scale ranging from 1 (*strongly disagree*) to 5 (*strongly agree*). Items relating to adaptive perfectionism (PS and O subscales) were excluded from analyses in the current study. Remaining items were grouped into a ‘parental’ maladaptive perfectionism score (total of PE and PC scores, ranging from 9 to 45) and an ‘intrapersonal’ maladaptive perfectionism score (total of CM and DA scores, ranging from 13 to 65), with higher scores representing greater maladaptive perfectionism. Consistent with previous research (Stallman, 2010), the FMPS had good overall reliability (α = 0.92) and internal consistency of subscales (α = 0.77 to 0.90) in the current study.

#### Internalized Shame Scale (ISS)

The ISS (Cook, 1988) is a 30-item measure assessing trait shame. Participants responded to a series of statements on a 5-point rating scale from 1 (*never*) to 5 (*always*). Of the total items, 24 were then summed to yield a ‘total shame’ score (ranging from 24 to 120), with higher scores indicating greater internalized shame. The remaining six items were excluded from analysis as they are adapted from the Rosenberg (1965) Self-Esteem Scale and included in the ISS to counteract response set bias. Instead, the full Rosenberg (1965) Self-Esteem Scale was included in the study as an independent measure of self-esteem. The ISS has demonstrated high temporal stability and internal consistency (α = 0.88 to 0.96) and adequate construct validity in clinical and non-clinical populations (Rybak and Brown, 1996; Del Rosario and White, 2006) and yielded a Cronbach’s alpha of (α = 0.97) in the current study.

#### Rosenberg Self-Esteem Scale (RSE)

The RSE (Rosenberg, 1965) is a self-report measure assessing global self-esteem. Participants responded to 10 items, five of which are positively worded (e.g., “on the whole, I am satisfied with myself”), and five negatively worded (e.g., “I certainly feel useless at times”) on a response scale from 1 (*strongly agree*) to 4 (*strongly disagree*). Positively worded items were reverse-coded, and all scores summed to give a total score ranging from 10 to 40, with higher scores indicating greater self-esteem (Rosenberg, 1965). The RSE has demonstrated good reliability and internal consistency (Goldsmith, 1986) and good construct validity in a range of cross-cultural samples (Schmitt and Allik, 2005). Cronbach’s alpha for the current study was (α = 0.88).

### Procedure

Data were collected for the study from two sources. ‘Clinical’ participants were student attendees of the University Psychology Clinic. Recruitment for the clinical sample was facilitated in-person by provisional psychologists on placement at the Psychology Clinic of the University, where the study took place, during initial assessment sessions, either through online or pen-and-paper means of completion. Besides, ‘non-clinical’ participant data was collected from students enrolled in first-year undergraduate psychology who took part in the study in return for course credit as part of a research experience program. Participants from the research experience program were asked to fill in an online questionnaire lasting approximately 30 min. All participants provided informed consent before completing the study. Ethics approval was gained from the university’s ethics committee.

### Data Analysis

Analyses were conducted in SPSS (IBM) v25.0 and MPlus. Between-group analyses were conducted to assess cross-cultural differences between Australian domestic and Asian international students on sociodemographic variables and study measures: *T*-tests were employed for continuous variables and chi-square tests for independence for categorical variables. Associations among studied variables were evaluated via bivariate correlations run separately for each group. Effect sizes of correlations were interpreted according to Cohen’s (1998) recommendations: small (*r* = 0.1), medium (*r* = 0.3), and large (*r* > 0.5).

**FIGURE 1:**
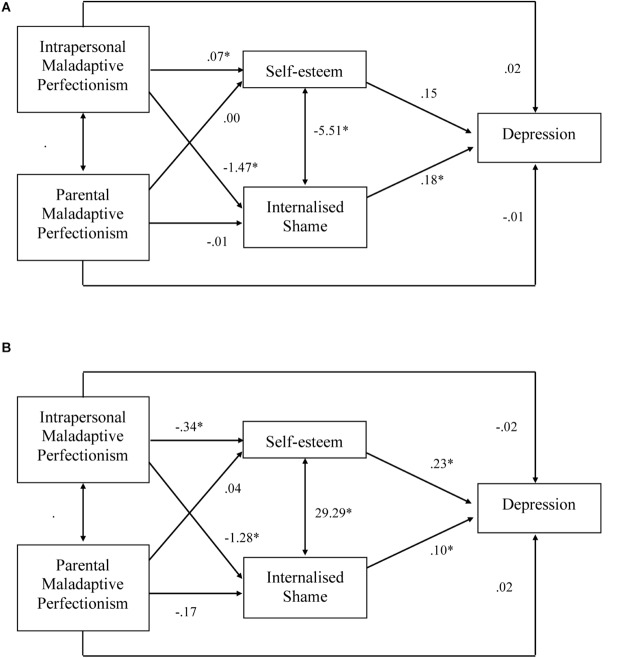
**(A)** Path model for Australian domestic sample. **(B)** Path model for Asian international students.

Path analysis was then employed to test the validity of the hypothesized model (Figure 1). Indirect effects in the model were tested for significance using accelerated bias-corrected bootstrapping with 5000 samples. This path model was run twice; once with parameter estimates allowed to vary across groups (i.e., for Australian domestic and Asian international students), and a second run in which parameters were constrained to be equal across the two groups. The plausibility of a single model for the two groups was evaluated by comparing model fit for the constrained and unconstrained path analyses. Two key statistics were used for this comparison: chi-square and the comparative fit index (CFI). A significant chi-square difference between the two models was used to identify statistically significant worsening of fit when forcing the model to be identical across groups, and a change in CFI values > 0.01 were taken to indicate worsening of model fit from a practical perspective (see Schermelleh-Engel et al., 2003).

For both the constrained and unconstrained models, fit was also assessed using chi-square, as well as CFI, root mean square error approximation (RMSEA), and standardized root-mean-square residual (SRMR). According to recommended standards for interpretation, good model fit was indicated by CFI values above 0.95, RMSEA below 0.10, and SRMR below 0.08 (Schermelleh-Engel et al., 2003). In the case of poor model fit, modification indices were consulted to identify the source of the misfit. Both models controlled for age and gender as known predictors of depression (Ibrahim et al., 2013).

## Results

### Sociodemographics

Sociodemographic and clinical characteristics of the sample are displayed in Table 1. The overall sample had a mean age of 20 years (*SD* = 4.38, range = 17–55 years) and was predominantly female (73.6%). As shown in Table 1, there were no significant differences in age or gender between domestic and Asian international students, or in any of the study variables except for parental criticism, which Australian domestic students reported at significantly higher levels then Asian international students; *t*(622) = 3.18, *p* = 0.002, Cohen’s *d* = 0.10. Slightly more than a third of the Asian international sample reported having been in Australia for less than a year (37%), while 24.3% reported having spent over 4 years in Australia.

**TABLE 1:** Descriptive statistics of the sample and main variables.

Variable	Asian international (*n* = 316)	Range of scores	Australian domestic (*n* = 308)	Range of scores	*p*	*χ*^2^/*t*
Mean age (*SD*)	19.75 (2.81)	17–39	20.22 (5.56)	17–55	0.185*	
Gender (%)		–		–	0.172	3.52
Male	86 (27.2)		79 (25.6)			
Female	230 (72.8)		229 (74.4)			
Intrapersonal maladaptive perfectionism	40.33 (10.23)	14–65	41.11 (10.36)	16–65	0.483	0.70
Parental maladaptive perfectionism	28.54 (7.79)	9–45	29.32 (7.76)	9–45	0.002	3.18
Depressive symptoms	12.53 (4.29)	7–28	12.81 (4.71)	7–26	0.433	0.78
Self-esteem	22.72 (4.85)	10–38	22.97 (2.08)	17–30	0.413	0.82
Internalized shame	61.16 (20.72)	24–118	61.22 (20.84)	24–118	0.973	0.03

### Correlational Analyses

Pearson’s correlations for study variables amongst each student group are presented in Table 2. According to Cohen’s (1988) recommendations, in both groups intrapersonal and parental maladaptive perfectionism demonstrated moderate to large significant positive correlations with each other and with self-esteem, and moderate to large significant negative correlations with depressive symptoms, and internalized shame. In both groups, internalized shame yielded strong significant positive correlations with depressive symptoms and moderate significant negative correlations with self-esteem. Interestingly, self-esteem only yielded small significant negative correlations with depressive symptoms amongst domestic students.

**TABLE 2:** Bivariate correlations between study variables.

	Intrapersonal MP	Parental MP	Depressive symptoms	Self-esteem	Internalized shame
Intrapersonal MP		0.48**	−0.52**	0.23**	−0.73**
Parental MP	0.54**		−0.27**	0.12**	−0.35**
Depressive symptoms	−0.51**	−0.27**		−0.09**	0.73**
Self-esteem	0.17**	0.11*	–0.03		−0.22**
Internalized shame	−0.67**	−0.40*	0.67**	−0.16**	

Correlations for the Asian international student sample are presented below the diagonal; correlations for the Australian domestic student sample are presented above the diagonal. * p < 0.05 (two-tailed). ** p < 0.01 (two-tailed). MP, maladaptive perfectionism.

### Path Analyses

The proposed model provided good fit for the data when parameter estimates were allowed to vary across groups; *χ^2^*(df = 8) = 10.692, *p* = 0.220, CFI = 0.997, RMSEA = 0.033, SRMR = 0.022. Forcing parameter values to be equal across groups led to a poor fitting model [*χ*^2^(df = 19) = 363.324, *p* < 0.001, CFI = 0.671, RMSEA = 0.241, SRMR = 0.167], and both significant [Δ*χ^2^*(df = 11) = 352.632, *p* < 0.001] and practical (ΔCFI = 0.326) worsening of model fit relative to the unconstrained model. Inspection of the modification indices suggested substantial differences across groups in the parameters for all relationships involving self-esteem. Given the breadth of differences across groups, we present separate model results for each group below instead of attempting a partial invariance model.

#### Australian Domestic Students

As shown in Figure 1A, depression was significantly and directly predicted by internalized shame. All other variables were not significant direct predictors of depression (*p*s ranged from 0.166 to 0.651). However, intrapersonal perfectionism had an indirect effect on depression via internalized shame (*b* = −0.258, 95% CIs: −0.313, −0.211). This relationship was not mediated by self-esteem. There was no mediated effect of parental perfectionism on depression via internalized shame or self-esteem.

Both self-esteem and internalized shame were significantly predicted by intrapersonal perfectionism but not parental perfectionism. Internalized shame and self-esteem were significantly related to each other. In total, the model accounted for 54, 11, and 54% of the variance in depressive symptoms, self-esteem, and internalized shame, respectively.

#### Asian International Students

Figure 1B shows significant relationships in the proposed model for Asian international students. Depression was significantly and directly predicted by internalized shame, self-esteem, and the covariate gender. All other variables were not significant direct predictors of depression (*p*s ranged from 0.526 to 0.609). However, the relationship between intrapersonal perfectionism and depression was significantly mediated by self-esteem (*b* = −.076, 95% CIs: −0.115, −0.039) and internalized shame (*b* = −0.125, 95% CIs: −0.172, −0.080). Parental perfectionism did not have a mediated effect on depression.

Both self-esteem and internalized shame were significantly predicted by intrapersonal perfectionism but not parental perfectionism. Shame and self-esteem were significantly related to each other. In total, the model accounted for 50, 46, and 45% of the variance in depressive symptoms, self-esteem, and shame, respectively.

## Discussion

This study is the first to examine and cross-culturally compare levels of, and relationships between, the intrapersonal and parental subtypes of maladaptive perfectionism, depression, self-esteem and internalized shame in samples of Australian domestic and Asian international university students. Findings revealed that the Australian domestic and Asian international student groups did not significantly differ on any study variables apart from maladaptive parental criticism: surprisingly Australian students reported higher levels of this than Asian university students. Furthermore, against expectations, the analysis revealed that higher intrapersonal and parental maladaptive perfectionism and higher internalized shame were significantly associated with lower levels of depressive symptoms. Finally, the proposed path model, in which maladaptive perfectionism subtypes lead directly to depressive symptoms as well as indirectly through both self-esteem and internalized shame was found to differ across Australian domestic and Asian international students, with fundamental differences in the models being related to the influence of self-esteem. Each of these findings will be discussed in further detail in subsequent sections.

### Cross-Cultural Differences in Levels of Study Variables

Contrary to expectations, Asian international students did not report significantly higher levels of depressive symptoms, internalized shame or parental or intrapersonal maladaptive perfectionism than the Australian domestic student sample. Indeed, Australian domestic students reported significantly *higher* levels of parental maladaptive perfectionism than their Asian international counterparts.

The lack of group differences on depressive symptoms between the student groups, challenges the classification of Asian international students as an ‘at-risk’ group in the tertiary student body for developing psychological distress such as depressive symptoms (e.g., Schofield et al., 2016). Instead, these findings support research that has found comparable rates of psychological distress across Australian domestic and Asian international university students (Khawaja and Dempsey, 2008). Similarly, the lack of significant difference in internalized shame between the student groups contradicts the notion that internalized shame is a variable particularly relevant to the distress of Asian international students (e.g., Anolli and Pascucci, 2005; Smith and Khawaja, 2011). Although not explicitly assessed in the current study, the lack of group differences on depressive symptoms and internalized shame may be related to culturally specific impacts of the self-report methodology of the study, as Asian samples have previously been found to underreport distress and associated psychological variables (e.g., Ang and Liamputtong, 2008). Future designs would benefit from the use of structured clinical interviews to more objectively assess levels of these study variables.

The finding that Australian domestic students reported significantly higher levels of parental maladaptive perfectionism relative to Asian international students is inconsistent with previous research, suggesting that maladaptive perfectionism is a particularly salient concern among Asian international students owing to a strong cultural focus on family obligations for academic achievements (e.g., Yoon and Lau, 2008). The current findings suggest that Australian domestic students may experience substantial family pressures to excel academically, perhaps at even greater levels than their Asian international student peers. It is currently unclear why this might be the case, as this study is the first to cross-culturally compare maladaptive perfectionism subtypes in Australian domestic and Asian international students. Thus, future replications are required to clarify these findings.

### Relationships Between Study Variables

Mixed support was found for the hypothesis that higher maladaptive perfectionism (both intrapersonal and parental types), lower self-esteem, and higher internalized shame would be significantly associated with higher depressive symptoms for both student groups.

#### Perfectionism and Depression

Contrary to expectations, intrapersonal and parental maladaptive perfectionism yielded both negative relationships with depressive symptoms in both Australian domestic and Asian international student groups. Across the literature, studies examining maladaptive perfectionism as a unitary construct have almost unanimously described positive associations with depressive symptoms (e.g., Egan et al., 2011; DiBartolo and Rendón, 2012). This is unsurprising, as the very nature of this form of perfectionism is defined by its ability to predict maladaptive outcomes such as anxiety and depression (e.g., Stoeber, 1998). However, some studies (e.g., Chang, 1998; Castro and Rice, 2003) examining the relationships between maladaptive perfectionism and psychopathology have reported mixed findings more in line with the current study. The current findings, therefore, suggest that parental and intrapersonal maladaptive perfectionism may relate similarly to depressive symptoms across the cultural groups assessed in the current study; however, further studies are required to verify these findings.

One possible explanation for the lack of a positive relationship between maladaptive perfectionism and depression in the current study may be the overall low levels of reported depressive symptoms (Khawaja and Dempsey, 2008). Alternatively, these findings may suggest the presence of intermediary moderating mechanisms between maladaptive perfectionism and depression that were not assessed in the current study, such as parental support (e.g., Seo, 2012) or perceived social support (e.g., Zhou et al., 2013), which have been found to alleviate the potential for distress generated by various forms of maladaptive perfectionism. It would be valuable for future research to explore the potential influence of these factors on the relationship between maladaptive perfectionism and depression.

#### Internalized Shame, Self-Esteem and Depression

As predicted, and in line with previous research, internalized shame was positively correlated with depressive symptoms (e.g., Kim et al., 2011), and negatively correlated with self-esteem (e.g., Velotti et al., 2017) for both Australian domestic and Asian international student groups. These findings support the conceptualization of internalized shame as a powerful cognition that negatively alters our self-image and can lead to considerable distress in individuals across cultures (e.g., Cook, 1988; Del Rosario and White, 2006).

Self-esteem yielded small negative correlations with depressive symptoms, but surprisingly, only the relationship amongst Australian domestic students was significant. This is somewhat conflicting with research that has consistently demonstrated self-esteem to be negatively related to depression across cultural groups such as Asian international students (e.g., Wei et al., 2007) and domestic Taiwanese adolescents (e.g., Lin et al., 2008), and American college students (e.g., Eisenbarth, 2012). Given that there is not yet unequivocal evidence regarding the causal direction of the relationship between self-esteem and depression (e.g., Sowislo and Orth, 2013), it is possible that the low levels of depressive symptoms reported in the current study made it less likely to yield significant relationships with reported levels of self-esteem.

### Testing the Proposed Path-Model

In line with the third hypothesis, the proposed path model, in which intrapersonal and parental maladaptive perfectionism lead to depressive symptoms via self-esteem and internalized shame, was not invariant (i.e., differed) across domestic and Asian international student groups. This finding is in line with other studies, which have found cross-cultural differences among Asian and Caucasian students on different types of depression models (e.g., Wei et al., 2007; Huang and Mussap, 2016), but contradicts other studies that have not been able to reveal such differences (e.g., Wang, 2007; Smith et al., 2017). For example, Smith et al. (2017), who conducted latent mean structure analysis on a large (*N* = 1006) sample of Chinese international and Canadian university students, found that perfectionism relating to depressive symptoms and well-being was invariant across student groups. Furthermore, Wang (2007) conducted a cross-cultural path-analysis study on a sample of 386 Caucasian-American and 252 Asian-American university students, finding that maladaptive perfectionism related to self-esteem and depression in a structurally invariant way across cultural student groups.

In the current study, results suggested that the principal sources of difference in the models between the student groups were in relationships involving self-esteem. Specifically, self-esteem directly predicted depressive symptoms and significantly mediated the relationship between intrapersonal perfectionism and depressive symptoms amongst only the Asian international student group. In both groups, internalized shame directly predicted depression and also mediated the relationships between intrapersonal perfectionism and depressive symptoms. Parental maladaptive perfectionism did not directly predict depressive symptoms nor indirectly predict depressive symptoms via internalized shame or self-esteem in either of the student groups.

The finding that self-esteem mediated the relationship between intrapersonal perfectionism and depressive symptoms among the Asian international student group, but not the domestic Australian student group, sheds important light on the cross-cultural nature of the processes by which different student groups develop depression from types of perfectionistic tendencies. These findings suggest that Asian international students who set high expectations for themselves and perpetually doubt their performance or worry about making mistakes may subsequently internalize perceived mistakes by experiencing an enduring sense of diminished self-concept, consequently leading to symptoms of depression. Based on these results, clinical interventions that specifically target intrapersonal maladaptive perfectionism and self-esteem through strategies such as psycho-education, cognitive restructuring and self-concept may be particularly helpful among Asian international students experiencing symptoms of depression.

In both Australian domestic and Asian international student groups, internalized shame mediated the relationship between intrapersonal maladaptive perfectionism and depressive symptoms. These findings suggest that shame may be a universally relevant mechanism by which intrapersonal maladaptive perfectionism leads to depression. Although this is the first study to examine the potential mediating role of internalized shame in the context of the subtypes of maladaptive perfectionism and depression, the current findings are consistent with Ashby et al. (2006). They reported that internalized shame mediated the relationship between a unitary measure of maladaptive perfectionism and depressive symptomatology. A more recent study (Elison and Partridge, 2012) among college athletes also found strong interrelationships between self-oriented perfectionistic tendencies, self-attacking and ruminating coping strategies, and internalization of shame experiences.

A further key finding of the current study was that against predictions, parental maladaptive perfectionism did not significantly predict depressive symptoms, either directly, or indirectly via self-esteem or internalized shame, in either student group. This is a valuable contribution to the literature, as to date, only one other study has examined the relationship between parental maladaptive perfectionism and depressive symptoms among university students (Yoon and Lau, 2008). In contrast to our findings, the study by Yoon and Lau (2008), parental (and intrapersonal) maladaptive perfectionism were both found to predict depressive symptoms among Asian American tertiary students strongly. Similarly, in another study (Wang et al., 2018), which assessed psychological distress (not depression) in Asian American and Asian international youngsters, the relationship between family perfectionism and psychological distress was mediated by family shame. Furthermore, ethnic group moderated the relationship between family perfectionism and family shame.

However, our finding is partially in agreement with a Taiwan-based study by Wang (2012), which found that composite scores of ‘personal’ perfectionism related more strongly to levels of self-esteem than did a composite of ‘perceived family perfectionism.’ Furthermore, in a validation study of the Chinese FMPS utilizing a large sample of 947 Chinese adolescents, concern over mistakes and doubt over actions (i.e., intrapersonal maladaptive perfectionism subscales) were found to predict the most significant variance in self-esteem above other subscales (Cheng et al., 2015).

It should also be noted that potential mediating mechanisms for the relationship between parental maladaptive perfectionism and depression were not assessed in the current study. For example, other studies have found variables such as parental support (Yoon and Lau, 2008; Seo, 2012) or self-silencing (Flett et al., 2007) to mediate or moderate this relationship. Yoon and Lau (2008) asserted that even when students perceive their parents as having high expectations and criticism over their performance, the development of internalizing symptoms (e.g., depression) will depend on whether students also perceive their parents as supportive (i.e., accepting, warm, etc.). Finally, it is also possible that in a university setting, these perfectionistic tendencies might be more powerful when they are being perceived to stem from a teacher, rather than the parents themselves. Accordingly, a study by Filippello et al. (2017) found psychological control from teachers, but not parents, to be linked to learned helpfulness, which is one of the core symptoms of depression, and this relationship was fully mediated by maladaptive perfectionism. Hence, future studies should include different sources (e.g., parents, peers, and teachers) of socially oriented perfectionism and related constructs (e.g., psychological control) in their mediating and/or moderating depression models.

Nonetheless, the current study’s finding that parent-focused maladaptive perfectionistic tendencies did not relate to depressive symptoms through internalized shame or self-esteem is significant. It is possible that unlike intrapersonal maladaptive perfectionistic tendencies, which seem to precipitate depressive symptoms through internalizing mechanisms; parental maladaptive perfectionism leads to depression through perceived external or environmental factors, such as perceived withdrawal of parental support (e.g., Yoon and Lau, 2008; Seo, 2012). However, it is only possible to speculate at this point, since other potential mediating mechanisms were not investigated in the current study.

### Limitations

It is important to recognize that the current study possessed several limitations. First, despite obtaining a large sample size with a relatively even spread of Asian international and domestic students, the fact that all respondents were students at one Australian university limits the generalizability of these findings to other university student populations. Second, we were not able to differentiate Asian international students in terms of their country of origin and their socio-economic backgrounds. Third, possible confounding factors associated with self-report data such as impression management, social desirability and perceived mental health stigma were not controlled for or assessed in the current study and may have biased responses. Finally, due to the cross-sectional design of this study, the findings obtained do not implicate causal links between variables.

### Recommendations for Future Research

Future studies should aim to extend on some of the findings in the current study while addressing some of its methodological limitations. For example, recruiting students from multiple universities may help to improve generalizability to the broader university student population. Future research may also benefit from employing more culturally sensitive and linguistically diverse measures (e.g., simplified Chinese character translation of the DASS; Chan et al., 2012), as some Asian international students may have been inadvertently excluded from participating in the current study due to linguistic barriers. Given the observed heterogeneity within the current sample of Asian international students (e.g., length of stay in Australia), future studies may benefit from including a measure of identification with Asian cultural values, such as the Asian Values Scale-Revised (AVS-R; Kim and Hong, 2004). It would also be helpful to include a measure that assesses explicitly perceived parental perfectionism, such as The Family Almost Perfect Scale (FAPS, Wang, 2010; Filippello et al., 2019). Examining the salience of these values within an Asian international student sample may help to explain the mechanism of potential cross-cultural differences in the variables assessed (Iwamoto and Liu, 2010). Future research should also aim to assess and control for possible factors influencing response bias particularly amongst Asian students, such as social desirability, impression management, and perceived mental health stigma (Smith, 2004; Eisenberg et al., 2009). The current study represents one of few that have examined maladaptive perfectionism in terms of parental and intrapersonal subtypes (Stoeber and Otto, 2006; Yoon and Lau, 2008). While path analyses in the current sample yielded exciting results in terms of the ways that each subtype may or may not relate to depressive symptoms, further research is needed to replicate these findings. Finally, conducting longitudinal research may also help to empirically establish how subtypes of maladaptive perfectionism may predispose students to develop depressive symptoms over time.

### Implications

Results from the current study suggest that maladaptive perfectionism may be a culturally dependent process, predisposing different groups of university students to develop depressive symptoms via different pathways. This, in turn, has implications for designing treatment programs aimed at preventing or alleviating depressive symptoms through alteration of maladaptive perfectionism. The current study also implies that maladaptive perfectionism may be a nuanced trait that should be treated as such in clinical practice with different groups of university students. If replicated, results from the current study suggest that clinicians should clarify whether students’ maladaptive perfectionistic traits are intrapersonal and should subsequently focus their interventions accordingly. For example, if a student demonstrated tendencies to doubt their performance or worry about making mistakes perpetually, this may warrant investigation and intervention around an internalized sense of shame, as this internalizing process may form a mechanism by which the student’s perfectionistic tendencies lead to depressive symptoms. Clinicians may also consider specifically targeting diminished self-concept among Asian international students. Although these implications for treatment are speculative at this stage, findings of the current study form a promising ground for future research aimed at improving the efficacy of perfectionism-focused treatments.

## Conclusion

In conclusion, the current study indicated no significant differences between Asian international and Australian domestic students on reported levels of depressive symptoms, self-esteem, internalized shame, and intrapersonal maladaptive perfectionism, however, Australian domestic students reported higher levels of parental maladaptive perfectionism. Besides, the hypothesized path model was found to differ across student groups. While intrapersonal maladaptive perfectionism led indirectly through internalized shame to depressive symptoms in both student groups, the indirect pathway from intrapersonal maladaptive perfectionism to depressive symptoms via self-esteem was only significant in the Asian international student group. The current findings implicate support for treating intrapersonal maladaptive perfectionism as a universal and culturally independent predisposing factor for the development of depressive symptoms amongst university students regardless of ethnicity or country of origin, however, suggest that self-esteem may be a particularly relevant mediating factor to target in treatment interventions amongst Asian international students. Also, the current findings support a nuanced conceptualization of maladaptive perfectionism, in which the intrapersonal dimension is particularly relevant to the development of psychological distress among university students. If replicated, this may support focused clinical interventions designed to treat intrapersonal maladaptive perfectionistic beliefs.

## Data Availability Statement

The datasets generated for this study are available on request to the corresponding author.

## Ethics Statement

All procedures performed in studies involving human participants were in accordance with the ethical standards of the institutional and/or national research committee and with the 1964 Helsinki declaration and its later amendments or comparable ethical standards. Informed consent was obtained from all individual participants included in the study.

## Author Contributions

BD, KB, and IK drafted the manuscript and conceptualized the aims and hypotheses. BD and MF-T conducted the analyses. BD, IK, and LP set up the data collection. All authors provided feedback on different versions of the manuscripts, read and approved the final manuscript and are accountable for all aspects of the work in ensuring that questions related to the accuracy of any part of the work are appropriately investigated.

## Conflict of Interest

The authors declare that the research was conducted in the absence of any commercial or financial relationships that could be construed as a potential conflict of interest.
